# MicroRNAs as novel peripheral markers for suicidality in patients with major depressive disorder

**DOI:** 10.3389/fpsyt.2022.1020530

**Published:** 2022-11-24

**Authors:** Britta Stapel, Ke Xiao, Nataliya Gorinski, Kevin Schmidt, Angelika Pfanne, Jan Fiedler, Imke Richter, Anna-Lena Vollbrecht, Thomas Thum, Kai G. Kahl, Evgeni Ponimaskin

**Affiliations:** ^1^Department of Psychiatry, Social Psychiatry and Psychotherapy, Hannover Medical School, Hanover, Germany; ^2^Fraunhofer Institute for Toxicology and Experimental Medicine (ITEM), Hanover, Germany; ^3^Fraunhofer Cluster of Excellence Immune-Mediated Diseases (CIMD), Hanover, Germany; ^4^Cellular Neurophysiology, Hannover Medical School, Hanover, Germany; ^5^Hannover Medical School, Institute of Molecular and Translational Therapeutic Strategies (IMTTS), Hanover, Germany

**Keywords:** major depressive disorder, suicidal ideation, suicide, microRNA, biomarker, early growth response protein 1 (EGR1)

## Abstract

**Objective:**

Major depressive disorder (MDD) constitutes a main risk factor for suicide. Suicide risk in psychiatric patients is primarily determined by often unreliable, self-reported information. We assessed serum levels of three microRNAs (miRNAs), previously demonstrated to be dysregulated in post-mortem brain samples of suicide victims, as potential peripheral biomarkers for suicidality.

**Methods:**

All study participants were diagnosed with MDD according to Diagnostic and Statistical Manual of Mental Disorders, 5th edition criteria. Suicidality, defined as acute suicide risk or suicide attempt within one week prior to study entry, was assessed by clinical interview. Relative serum levels of miR-30a, miR-30e, and miR-200a, normalized to U6, were measured by quantitative real-time PCR in MDD inpatients with (MDD/SI, *N* = 19) and without (MDD, *N* = 31) acute suicide risk. Median age and gender distribution were comparable in both groups.

**Results:**

Levels of miR-30a, miR-30e, and miR-200a were significantly elevated in MDD/SI compared to MDD. Subgroup analysis of the MDD/SI group showed that levels of miR-30e and miR-200a were significantly higher and miR-30a was increased by trend in patients admitted following a suicide attempt (*N* = 7) compared to patients with acute suicide risk but without recent suicide attempt (*N* = 12). Additionally, use of two databases for *in silico* transcription factor–miRNA interaction prediction indicated early growth response protein (EGR) 1 as potential transcriptional regulator for all three miRNAs.

**Conclusion:**

This study demonstrates suicide risk in MDD patients to be associated with increased levels of miR-30a, miR-30e, and miR-200a. Thus, these miRNAs might constitute potential biomarkers to predict suicidal behavior in MDD patients.

## Introduction

Major depressive disorder (MDD), defined as at least one discrete depressive episode that lasts for more than two weeks, is a debilitating mental disorder that is estimated to affect about 6% of the adult population worldwide every year (1). When measured as “years lived with disability,” MDD constitutes the second leading burden of disease (2). Importantly, MDD is one of the main risk factors for death by suicide. According to the World Health Organization, every year over 800,000 people die due to self-inflicted injuries (3). This accounts for 1.5% of total death and makes suicide one of the leading causes of death in people 15–34 years of age (4). In this regard, it is also noteworthy that the number of non-fatal suicide attempts significantly exceeds the number of suicides, i.e., for each death by suicide, there are an estimated 25 suicide attempts (5, 6). In the context of MDD, results from the ESEMED study, a European cross-sectional household survey, indicate that attributable risk for lifetime suicide attempt was 28% for MDD and additionally, major depressive episode together with alcohol dependency determined most strongly the progression from suicidal ideation to suicide attempt (7).

Currently, clinicians determine suicide risk in psychiatric patients primarily based on information regarding prior suicidal behavior and a family history of suicide as provided by patients. However, reliability of self-reported information concerning suicidal ideation is limited (8) and information regarding the family history of suicidal behavior might be inaccurate due to the associated stigma.

Given the significant personal as well as economic impact that suicide imposes especially in patients with MDD, reliable markers to objectively identify suicide risk are required to improve diagnosis, classifications and outcomes.

The etiology of MDD and suicidality is currently considered to involve a complex interplay of genetic and environmental factors (9). Accordingly, research targeting the identification of potential biomarkers for suicide risk in patients with MDD has focused on structural and functional brain alterations assessed by brain imaging as well as on biochemical and genomic markers associated with systems implicated in depression and suicidal behavior (10). However, based on the well-established association of early as well as of acute adverse life events with MDD and suicide risk (11–16), epigenetic markers have recently come into focus (17). While epigenetic mechanisms are classically defined as modulations of DNA organization in form of DNA methylation and histone modifications (18–20), more recently, non-coding RNAs have been identified as post-transcriptional regulators that mediate stress-induced changes in the context of psychiatric disorders (20). Within the group of non-coding RNAs, microRNAs (miRNAs) were first described as single-stranded RNA molecules with a length of 19–25 base-pairs that bind to 3’-untranslated regions (3’UTRs) of their respective target mRNAs resulting in repression of translation or alterations in mRNA stability (21–23). Since then, various studies have investigated the involvement of miRNAs in the etiopathology of psychiatric disorders including MDD and suicidal ideation (24). Importantly, alterations in miRNA levels are not limited to the brain but distinct changes were also detected in blood samples of depressed patients highlighting their potential as putative biomarkers for MDD and suicidal behavior (24, 25).

We previously described alterations in miR-30a and miR-30e as well as in miR-200a levels in post-mortem brain samples of individuals with MDD that died by suicide compared to mentally healthy individuals that died of unrelated causes (26). Given the high prevalence of suicidality in MDD patients and the need for reliable biomarkers to determine suicide risk, we aimed to assess whether these observed dysregulations in miRNA expression could be replicated in a pilot study with depressed patients with and without suicidality and suicide attempt.

## Materials and methods

### Subjects

The present study was performed in accordance with principles expressed in the Declaration of Helsinki and was approved by the local ethics committee at Hannover Medical School. Written, informed consent was given by all participants before study entry. Fifty patients diagnosed with MDD in accordance to the Diagnostic and Statistical Manual of Mental Disorders 5th edition (DSM-V) criteria were included in the study. All patients were treated in the Department of Psychiatry, Social Psychiatry and Psychotherapy at Hannover Medical School at the time of study entry.

Suicidality, defined as acute suicide risk or suicide attempt within 1 week prior to study entry, was determined as follows: (1) patients self-report of acute suicidality, (2) clinician’s impression of acute suicidality, (3) necessity of psychiatric intensive care unit (PICU) treatment (either patients were transferred to PICU internally after admission following a suicide attempt, or were directly admitted to PICU as emergencies from outside). Nineteen depressed patients fulfilled the criterion suicidality (MDD/SI). Of these, *N* = 12 patients were at acute suicide risk, and *N* = 7 patients were assessed following a suicide attempt. Blood of these patients was drawn within 1 week of admission to the clinics. Thirty-one depressed inpatients without the criterion suicidality according to clinical impression and chart analysis were included as a control group (MDD).

Exclusion criteria for both the both groups included current or lifetime substance use disorder (excluding nicotine), schizophrenia, bipolar disorder, eating disorders, intellectual disability, any acute physical disorder, in particular current or lifetime (auto-) immune disease, type-2 diabetes mellitus, cardiac diseases, systemic diseases, and current infectious disease and an age < 18 years.

### Blood sampling and miRNA measurement

Blood was drawn under fasting conditions. Samples were collected in serum S-Monovettes (Sarstedt, Nümbrecht, Germany), centrifuged at 2,000 rpm for 15 min. Finally, 200 μl of blood serum was transferred into fresh tube and stored at –80°C. Isolation of miRNAs from samples was performed using the miRNeasy Serum/Plasma Advanced Kit (Qiagen, Hilden, Germany) according to manufacturer’s protocol and miRNAs were eluted in water. Reverse transcriptions were performed with the TaqMan™ MicroRNA Reverse Transcription Kit. (Applied Biosystems, Waltham, MA, USA). Subsequently, quantitative real time polymerase chain reaction (qPCR) of miR-30a, miR-30e and miR-200a was conducted with respective TaqMan™ Assays (hsa-miR-30a-5p, ID 000417; hsa-miR-30e, ID 002223; hsa-miR-200a, ID 000502; all Thermo Scientific, Waltham, MA, USA) using the ABsolute Blue QPCR Mix (Thermo Scientific, Waltham, MA, USA). Additionally, U6 snRNA levels were measured (ID: 001973, Thermo Scientific) for expression level normalization. All qPCRs were run on a QuantStudio™ 7 Flex System (Applied Biosystems, Waltham, MA, USA) amplification and detection machine. Results were analyzed with the QuantStudio™ Real-Time PCR Software v1.3 (Applied Biosystems, Waltham, MA, USA). In corresponding graphs, 2^–ΔCt^ values are displayed.

### Prediction of transcriptional regulators of miRNAs and of potential miRNA targets

TransmiR v2.0 database (27) and the RegNetwork database (28) were used individually to predict transcription factors (TFs) that can putatively regulate the three miRNAs investigated in this study. Additionally, Targetscan was used to identify potential target genes of the assessed miRNAs (29).

### Statistical analyses

All statistical analyses were performed using SPSS version 26 (IBM, Armonk, NY, USA). Extreme outliers defined as values three times the interquartile range (IQR) were excluded from the respective analyses. Kolmogorov-Smirnoff test was used to test for normal distribution of data and subsequently, Mann-Whitney U test was applied to assess group differences. To analyze group differences of nominal data, Chi Square test followed by Fisher’s Exact test was utilized. Two-tailed *P*-values are depicted and *P*-values < 0.05 were considered statistically significant.

## Results

### Demographics, clinical variables, and medication of the major depressive disorder/SI compared to the major depressive disorder group

No significant differences with regard to age (*U* = 233.00, *Z* = –1.230, *P* = 0.223) and gender distribution [χ^2^(1) = 0.002, *P* = 1.00] were observed between the MDD/SI and the MDD group (Table 1).

**TABLE 1:** Anthropometric data and psychopharmacologic drug treatment in the MDD and the MDD/SI group.

	MDD (*N* = 31)	MDD/SI (*N* = 19)	Statistics
**Anthropometric data**			
Age [median (IQR) in years]	33 (23–48)	29 (23–37)	*U* = 233.00, *Z* = –1.230, *P* = 0.223 a
Female gender [*N* (%)]	21 (68%)	13 (68%)	χ^2^(1) = 0.002, *P* = 1.00 b
**Comorbidities**			
BPD [*N* (%)]	11 (36%)	9 (47%)	χ^2^(1) = 0.552, *P* = 0.555 b
PTSD [*N* (%)]	1 (3%)	7 (37%)	χ^2^(1) = 9.561, *P* = 0.004 b
**Psychopharmacologic drugs**			
None [*N* (%)]	8 (26%)	0 (0%)	χ^2^(1) = 5.837, *P* = 0.018 b
SSRI [*N* (%)]	8 (26%)	4 (21%)	χ^2^(1) = 0.146, *P* = 0.748 b
SNRI [*N* (%)]	9 (29%)	4 (21%)	χ^2^(1) = 0.390, *P* = 0.742 b
NDRI [*N* (%)]	4 (13%)	4 (21%)	χ^2^(1) = 0.581, *P* = 0.459 b
Agomelatine [*N* (%)]	5 (16%)	3 (16%)	χ^2^(1) = 0.001, *P* = 1.00 b
Mirtazapine [*N* (%)]	1 (3%)	7 (37%)	χ^2^(1) = 9.905, *P* = 0.003 b
Atypical antipsychotic [*N* (%)]	0 (0%)	10 (53%)	χ^2^(1) = 20.395, *P* < 0.001 b
Lithium [*N* (%)]	0 (0%)	2 (11%)	χ^2^(1) = 3.399, *P* = 0.140 b
Pipamperone [*N* (%)]	2 (7%)	7 (37%)	χ^2^(1) = 7.371, *P* = 0.018 b
Benzodiazepine [*N* (%)]	1 (3%)	2 (11%)	χ^2^(1) = 1.113, *P* = 0.549 b
Methylphenidate [*N* (%)]	1 (3%)	0 (0%)	χ^2^(1) = 0.625, *P* = 1.00 b
Trazodone [*N* (%)]	0 (0%)	1 (5%)	χ^2^(1) = 1.665, *P* = 0.380 b
Sedative [*N* (%)]	0 (0%)	6 (32%)	χ^2^(1) = 11.124, *P* = 0.002 b

Exact, two-tailed *P*-values are depicted and group comparisons that reached statistical significance are highlighted in gray. BPD, borderline personality disorder; IQR, interquartile range; NDRI, norepinephrine-dopamine reuptake inhibitor; PTSD, posttraumatic stress disorder; SNRI, serotonin-norepinephrine reuptake inhibitor; SSRI, selective serotonin reuptake inhibitor. a: Mann-Whitney U test. b: Chi-squared test with Fisher’s Exact test.

Relevant psychiatric comorbidities included borderline personality disorder (BPD) that was similar in frequency in the MDD/SI and in the MDD group [χ^2^(1) = 0.552, *P* = 0.555] and posttraumatic stress disorder (PTSD) that was more frequent in the MDD/SI group than in the MDD group [χ^2^(1) = 9.561, *P* = 0.004] (Table 1).

Concerning psychopharmacologic drug treatment, all patients in the MDD/SI group [*N* = 19 (100%)], and 23 out of 31 patients in the MDD group (74.2%) received at least one psychopharmacologic drug [χ^2^(1) = 5.837, *P* = 0.018] (Table 1). In this regard, use of mirtazapine [χ^2^(1) = 9.905, *P* = 0.003], of pipamperone [χ^2^(1) = 7.371, *P* = 0.018], and of atypical antipsychotics [χ^2^(1) = 20.395, *P* < 0.001] was more frequent in the MDD/SI group when compared to the MDD group (Table 1). Finally, *N* = 6 patients in the MDD/SI group received a sedative, compared to none of the patients in the MDD group [χ^2^(1) = 11.124, *P* = 0.002] (Table 1).

### Relative miRNA levels in the major depressive disorder/SI compared to the major depressive disorder group

**FIGURE 1:**
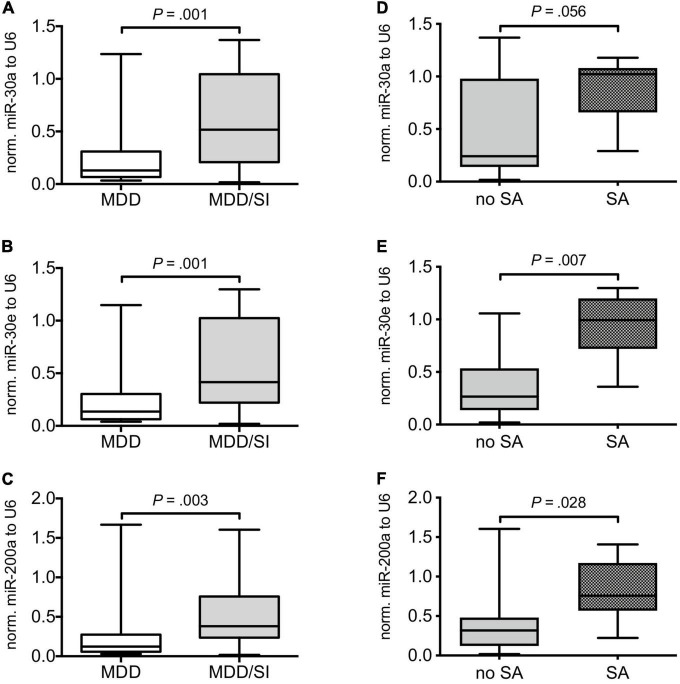
Comparison of relative miRNA expression in MDD patients with and without acute suicide risk and in suicidal MDD patients with and without suicide attempt. Boxplots depict median with interquartile range and whiskers depict minimum and maximum values. The left panel shows relative miR-30a **(A)**, miR-30e **(B)**, and miR-200a **(C)** expression normalized to U6 in MDD patients without (MDD) and in MDD patients with (MDD/SI) acute suicide risk. The right panel depicts relative levels of miR-30a **(D)**, miR-30e **(E)**, and miR-200a **(F)** in suicidal patients with (SA) and without (no SA) suicide attempt. Exact, two-tailed *P*-values were computed by use of Whitney-Mann U test and *P* < 0.05 was considered statistically significant.

Relative serum levels of miR-30a (*U* = 123.00, *Z* = –3.235, *P* = 0.001), miR-30e (*U* = 114.00, *Z* = –3.223, *P* = 0.001) and of miR-200a (*U* = 150.00, *Z* = –2.888, *P* = 0.003) normalized to U6 were significantly higher in the MDD/SI group compared to the MDD group (Figures 1A–C and Supplementary Table 1).

### Demographics, clinical variables, and medication of major depressive disorder/SI subgroups based on recent suicide attempt

As the MDD/SI group displayed heterogeneous values with regard to miRNA expression, sub-group analyses of the MDD/SI group were performed to compare suicide attempters (SA) to individuals with an acute suicide risk, but without suicide attempt (no SA). Both subgroups were comparable regarding mean age (*U* = 31.00, *Z* = –0.931, *P* = 0.372) and gender distribution [χ^2^(1) = 0.046, *P* = 0.622] (Table 2). Additionally, no significant differences in psychopharmacological medication were present (Table 2). However, comorbid BPD tended to be more frequent in the no SA subgroup [χ^2^(1) = 4.866, *P* = 0.057], while frequency of PTSD diagnosis was comparable between the SA and the no SA subgroups [χ^2^(1) = 0.326, *P* = 0.656] (Table 2).

**TABLE 2:** Anthropometric data and psychopharmacologic drug treatment in MDD/SI subgroups with and without suicide attempt (SA).

	No SA (*N* = 12)	SA (*N* = 7)	Statistics
**Anthropometric data**			
Age [median (IQR) in years]	27 (20–44)	31 (26–37)	*U* = 31.00, *Z* = –0.931, *P* = 0.372 a
Female gender [*N* (%)]	8 (67%)	5 (71%)	χ^2^(1) = 0.046, *P* = 0.622 b
**Comorbidities**			
BPD [*N* (%)]	8 (67%)	1 (14%)	χ^2^(1) = 4.866, *P* = 0.057 b
PTSD [*N* (%)]	5 (42%)	2 (29%)	χ^2^(1) = 0.326, *P* = 0.656 b
**Psychopharmacologic drugs**			
SSRI [*N* (%)]	2 (17%)	2 (29%)	χ^2^(1) = 0.377, *P* = 0.603 b
SNRI [*N* (%)]	3 (25%)	1 (14%)	χ^2^(1) = 0.305, *P* = 1.00 b
NDRI [*N* (%)]	3 (25%)	1 (14%)	χ^2^(1) = 0.305, *P* = 1.00 b
Agomelatine [*N* (%)]	2 (17%)	1 (14%)	χ^2^(1) = 0.019, *P* = 1.00 b
Mirtazapine [*N* (%)]	3 (25%)	4 (57%)	χ^2^(1) = 1.963, *P* = 0.326 b
Atypical antipsychotic [*N* (%)]	7 (58%)	3 (43%)	χ^2^(1) = 0.425, *P* < 0.650 b
Lithium [*N* (%)]	1 (8%)	1 (14%)	χ^2^(1) = 0.166, *P* = 1.00 b
Pipamperone [*N* (%)]	5 (42%)	2 (29%)	χ^2^(1) = 0.326, *P* = 0.656 b
Benzodiazepine [*N* (%)]	1 (8%)	1 (14%)	χ^2^(1) = 0.166, *P* = 1.00 b
Trazodone [*N* (%)]	0 (0%)	1 (14%)	χ^2^(1) = 1.810, *P* = 0.368 b
Sedative [*N* (%)]	4 (33%)	2 (29%)	χ^2^(1) = 0.046, *P* = 1.00 b

Exact, two-tailed *P*-values are depicted. BPD, borderline personality disorder; IQR, interquartile range; NDRI, norepinephrine-dopamine reuptake inhibitor; PTSD, posttraumatic stress disorder; SA, suicide attempt; SNRI, serotonin-norepinephrine reuptake inhibitor; SSRI, selective serotonin reuptake inhibitor. a: Mann-Whitney U test. b: Chi-squared test with Fisher’s Exact test.

### Relative miRNA levels in of major depressive disorder/SI subgroups based on recent suicide attempt

Subsequent subgroup analyses of the MDD/SI group showed significantly higher values of miR-30e (*U* = 8.00, *Z* = –2.635, *P* = 0.007) and of miR-200a (*U* = 16.00, *Z* = –2.197, *P* = 0.028) in SA patients when compared to the no SA group. Additionally, a trend toward higher values of miR-30a was observed in the SA group compared to the no SA group (*U* = 17.00, *Z* = –1.947, *P* = 0.056) (Figures 1D–F and Supplementary Table 2).

### Database analyses of transcription factor binding in miRNA promoter regions

**FIGURE 2:**
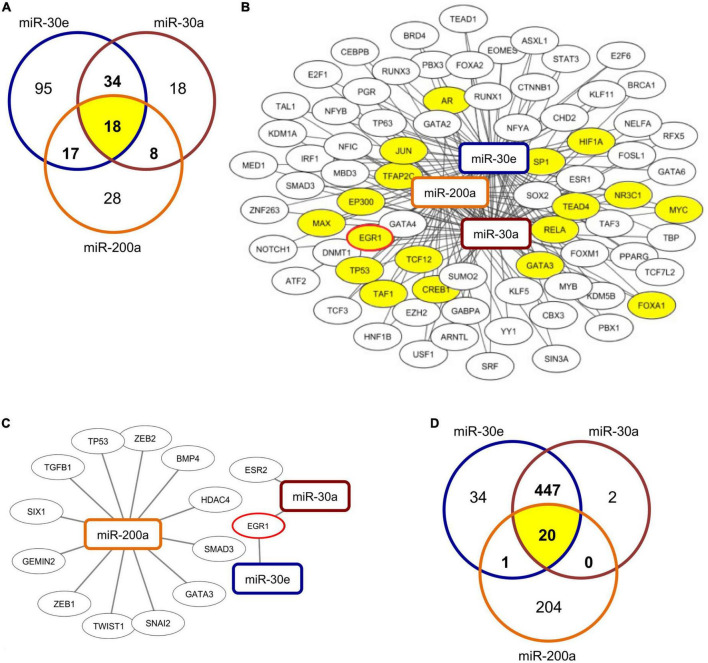
Prediction of TF-miRNA binding and of potential miRNA targets. **(A)** Venn diagram depicts number of predicted transcription factors (TF) that are able to interact with the respective miRNAs and their overlap and **(B)** shows network of TF-miRNA interactions of 77 TFs predicted to interact with at least two of the three assessed miRNAs. Visualization was carried out using the Cytoscape software with the respective miRNAs predicted by TransmiR database. The 18 common TFs predicted to interact with all three miRNAs are highlighted in yellow. **(C)** Network of predicted TF-miRNA interactions from RegNetwork database analysis. 14 TFs were predicted to interact with at least one of the assessed miRNAs. Visualization was carried out by use of the Cytoscape software. **(D)** Venn diagram depicts number of predicted targets of respective miRNAs and their overlap.

To identify potential transcriptional regulators of the differentially expressed miRNAs, miR-30a, miR-30e, and miR-200a were submitted to the TransmiR v2.0 database (27) to predict putative transcription factor (TF) binding sites within their respective promoter regions. The approach indicated 77 TFs that were predicted to putatively regulate at least two of the three miRNAs, and 18 TFs, including early growth response 1 (EGR1), that were implicated in the regulation of all three miRNAs (Figures 2A,B). To confirm the obtained results, RegNetwork as a second TF-miRNA database was utilized in parallel (28). Using this approach, 14 TFs were identified to be able to regulate one of the three miRNAs with high confidence (Figure 2C and Supplementary Table 3). Among them, EGR1 was found to putatively interact with miR-30a and miR-30e. Overall, comparing results from both approaches, EGR1 was predicted as a potential transcriptional regulator of the relevant miRNAs with high confidence.

### *In silico* analyses of predicted miRNA targets

Targetscan (29) was used to identify potential target genes of the assessed miRNAs. *In silico* analyses identified 469 genes that can potentially be targeted by miR-30a, 502 genes that are potential targets of miR-30e, and 225 genes that constitutes potential targets of miR-200a (Figure 2D) at the 3′UTR region. Among them 20 genes were predicted to be targeted by all three miRNAs (Supplementary Table 4).

## Discussion

In the present study, we report significantly higher relative expression levels of three miRNAs (i.e., miR-30a, miR-30e, and miR-200a) in blood serum samples from suicidal MDD patients compared to an MDD group that did not display current suicide risk. It is noteworthy that our previous study demonstrated that expression of these miRNAs was dysregulated in post-mortem brain samples of MDD patients that died by suicide (26). Additionally, we propose transcription factor EGR1 as potential regulator of miRNA expression.

Studies dedicated to the identification of suitable biomarkers for MDD and therapeutic responses to psychopharmacologic treatment are numerous and subsequently potential molecular markers have been identified on the genomic, epigenomic, transcriptomic, and proteomic level (30, 31).

Contrarily, studies that aim to identify markers for suicidality are less frequent. In this regard, several studies assessed alterations in miRNA expression in different brain regions of suicide victims (32, 33). However, while these findings offer important insights in potential pathomechanisms underlying suicidal behavior, interpretation of results is complicated by an often unclear psychiatric diagnose and/or by the lack of an appropriate control group (32). Consequently, post-mortem studies often do not allow for a differentiation between miRNA regulation linked to suicidality and changes brought forward by the underlying psychiatric illness. Therefore, a replication of post-mortem results in clinical samples is of significant importance.

In the context of central nervous system (CNS)-related disorders, including psychiatric disorders and MDD, numerous studies have described alterations in miRNA levels in peripheral blood samples (25), and it has been shown that alterations in brain miRNA expression appear to be reflected in the periphery (34, 35). Albeit the need for reliable biomarkers that objectively predict suicide risk in MDD patients and the demonstrated potential of peripheral miRNAs as suitable biomarkers in the context of CNS-related diseases (34), a recent systemic review identified only two studies that assessed miRNAs as potential biomarkers for suicidality in peripheral blood samples (33) and neither study included patients with recent suicide attempt (36, 37).

This is of importance, as the observed higher levels of miR-30a, miR-30e, and miR-200a in suicidal MDD patients compared to those without current suicide risk in this study, could be mostly attributed to a subgroup of patients that had recently attempted suicide compared to those without a recent suicide attempt.

Our observation is in line with the understanding that suicidal behavior, defined as suicide attempt or suicide completion, is characterized by overlapping, psychobiological abnormalities that are discernable from suicidal ideation (38). Accordingly, it is generally thought that vulnerability to suicidal behavior is mediated partly by an underlying genetic predisposition, which interacts with environmental and probable epigenetic factors (39), and might be reflected by changes in miRNA expression. Indeed, several studies demonstrated changed miRNA expression profiles in post-mortem as well as peripheral blood samples from MDD patients (40, 41). In particular, our previous study identified altered expression levels of miR-30a, miR-30e, and miR-200a in prefrontal cortex (PFC) samples of suicide victims diagnosed with depression compared to mentally healthy controls that died of unrelated causes (26). Interestingly, an association analyses in MDD patients and control subjects identified polymorphisms within the miR-30e precursor to be positively correlated with depression and its symptomatic onset (42). Moreover, results from a social defeat stress mouse model indicated a downregulation of miR-30a and miR-30e in the context of stress and depression-like behavior and in hippocampal neurogenesis (43). Intriguingly, we have recently found that the higher prevalence for MDD and panic disorders in women with peripartum cardiomyopathy was associated with elevated levels of miR-30e (44). Finally, alterations in miR-200a expression have been reported in a rodent model of unpredictable chronic mild stress as well as in plasma samples of patients with Alzheimer’s disease (45–47).

Regarding potential upstream regulators of miR-30a, miR-30e, and miR-200a transcription, predictions based on two independent database searches identified EGR1 as a potential transcriptional regulator of the assessed miRNAs. EGR1, also known as NGFI-A, constitutes a nuclear phosphoprotein attributed with an activation potential as well as with a negative regulatory function (48–51). Among others, EGR1 was found to be critically involved in neuroplasticity (52). Decreased EGR1 expression was found in post-mortem PFC samples of MDD patients in comparison to healthy individuals (53) and its downregulation was also detected in animal models of depression and anxiety in specific brain areas including the hippocampus (53, 54). Importantly, a study based on transcriptome sequencing data derived from post-mortem PFC samples found a gradual downregulation of a gene cluster that included EGR1 in non-suicidal MDD patients compared to control with a further decrease being detected in suicidal MDD patients (55). Given the opposite regulation of EGR1 (i.e., decreased in the context of MDD and suicidality) and of the miRNAs assessed in this study (i.e., increased in the context of SI and SA), one might speculate that EGR1 could function as a negative regulator of these miRNAs. In this regard, repressor function of EGR1 has been previously shown in neuronal cells (56–58) and positive as well as negative regulatory effects of EGR1 on miRNA expression have been described in various tissues and disease states (59–63) and EGR1 binding sites within the predicted promoter region of miR-30a and miR-30e were identified (64). A potential transcriptional regulation of the relevant miRNAs by EGR1 and its direction should be concern of future studies.

Concerning potential targets of miRNA-30a, miR-30e, and miR-200a, *in silico* analysis identified 20 mRNAs that were predicted to be regulated by all three miRNAs via their respective 3’ UTRs. To our knowledge, out of these predicted targets, only three [i.e., muscleblind like splicing regulator 1 (MBNL1), receptor accessory protein 3 (REEP3), retinoic acid receptor beta (RARB)] have previously been reported in the context of depression or depression-like phenotypes in the literature and none were described to be associated with suicidality (65–68). In this regard, only RARB has been reported in the context of MDD in humans (68) and rodents (66), while MBNL1 and REEP3 were reported only in rodent models (65, 67). The respective studies utilized screening approaches and we are unaware of studies that have investigated the function of the respective genes in the context of depression and/or suicidality. While our *in silico* analysis gives a first indication of potential targets of the assessed miRNAs in the context of depression and suicidality, further studies are needed to confirm these targets *in vitro* and to assess their respective relevancy in the context of suicidality in patients with MDD.

In conclusion, we found that miRNAs previously described to be dysregulated in post-mortem brain samples of MDD patients that died by suicide, were elevated in serum samples of MDD patients that displayed current suicide risk in comparison to MDD patients that did not show acute suicidal ideation. Our results suggest that the observed miRNAs might function as a trait marker of suicidal behavior (i.e., suicide attempt and suicide completion) as the overall higher levels could be mainly attributed to a patient subgroup that attempted suicide in the week prior to study entry. Therefore, further studies might focus not only on the potential application of these miRNAs as biomarkers for suicidal behavior, but also on their respective involvement in the pathomechanisms that underlie suicidal behavior in general and in the context of MDD in particular.

### Limitations

This pilot study has several limitations. Firstly, we do not provide data from a second validation cohort. Additionally, the respective MDD groups in this study are heterogeneous regarding psychopharmacological treatment and psychiatric comorbidities. This is of importance as pharmacological drugs might differentially impact miRNA expression at the subcellular level which we also cannot specify herein (69). Next to intracellular miRNA biogenesis, we can only speculate on miRNA secretion to patient’s plasma, which is often a directed mechanism via extracellular vesicles (70). Additionally, it remains unclear whether the neurobiology of suicidality is consistent across different diagnoses. For instant, an imaging study found mGluR5 availability to be associated with suicidality in patients with PTSD but not in those with MDD (71). On the other hand, it has also been demonstrated that suicidal behavior (suicide attempt and completion) are characterized by psychobiological abnormalities that are independent of co-occurring psychiatric disorders (72). As PTSD was more frequent in the MDD/SI group compared to the MDD group, we cannot exclude that PTSD comorbidity contributed to the observed effects. However, given the fact that frequency of PTSD comorbidity as well as medication did not significantly differ in the respected MDD/SI subgroups it appears likely that the observed differential miRNA levels are indeed brought forward by suicidal behavior. Finally, identification of suicidal ideation might lack accuracy as its diagnosis in our study solely relied on self-report in the MDD/SI group, and on chart analysis in the MDD group without suicidality. This might be of importance as it has been shown that prior suicide attempts partly predict future suicidality (73).

## Data availability statement

The original contributions presented in this study are included in the article/Supplementary material, further inquiries can be directed to the corresponding author/s.

## Ethics statement

The studies involving human participants were reviewed and approved by Ethics Committee, Hanover Medical School. The patients/participants provided their written informed consent to participate in this study.

## Author contributions

BS: formal analysis and writing—original draft. KX and NG: investigation, formal analysis, methodology, and visualization. KS: investigation and formal analysis. AP: methodology and writing—review and editing. JF: supervision, methodology, and writing—review and editing. IR and A-LV: resources. TT, KK, and EP: supervision, funding acquisition, project administration, conceptualization, formal analysis, and writing—review and editing. All authors contributed to the article and approved the submitted version.
