# Supplementary material for: MicroRNAs as novel peripheral markers for suicidality in patients with major depressive disorder
Source: Front Psychiatry. 2022 Nov 24;13:1020530. doi: 10.3389/fpsyt.2022.1020530 (PMC9729747; doi:10.3389/fpsyt.2022.1020530)
Supplement: Supplementary file 1 [file Data_Sheet_1.PDF]

# *Supplementary Material*

## 1 Supplementary Data

This file contains two supplementary tables detailing relative expression levels of miR-30a, miR-30e, and miR-200a in the respective MDD groups and in the MDD/SI subgroups. A third supplementary table depicts results from the RegNetwork database analysis concerning TF-miRNA interactions and a fourth supplementary table shows common target genes of miR-30a, miR-30e, and miR-200a predicted by Targetscan.

## 2 Supplementary Tables

**Supplemental Table 1: Comparison of relative miRNA expression levels in the MDD and MDD/SI group.**

| | Group | *N* | Median | IQR | Whitney-Mann U test |
|---|---|---|---|---|---|
| miR-30a | MDD | 31 | .130 | .067-.309 | *U*=123.00, *Z*=-3.235, *P*=.001 |
| | MDD/SI | 18 | .517 | .208-1.044 | |
| miR-30e | MDD | 31 | .135 | .063-.303 | *U*=114.00, *Z*=-3.223, *P*=.001 |
| | MDD/SI | 17 | .415 | .221-1.025 | |
| miR-200a | MDD | 31 | .123 | .058-.275 | *U*=150.00, *Z*=-2.888, *P*=.003 |
| | MDD/SI | 19 | .381 | .236-.758 | |

Whitney-Mann U test followed by Fisher's Exact test was applied to assess group differences. Two-tailed *P*-values are depicted and comparisons that were statistically significant are highlighted in gray. MDD, major depressive disorder; IQR, interquartile range; SI, suicidal ideation.

**Supplemental Table 2: Comparison of miRNA expression levels in the MDD/SI subgroups with and without suicide attempt (SA).**

| | Group | *N* | Median | IQR | Whitney-Mann *U* test |
|---|---|---|---|---|---|
| miR-30a | no SA | 11 | .242 | .139-.979 | *U*=17.00, *Z*=-1.947, *P*=.056 |
| | SA | 7 | 1.002 | .658-1.081 | |
| miR-30e | no SA | 10 | .266 | .137-.534 | *U*=8.00, *Z*=-2.635, *P*=.007 |
| | SA | 7 | .993 | .720-1.200 | |
| miR-200a | no SA | 12 | .318 | .121-.482 | *U*=16.00, *Z*=-2.197, *P*=.028 |
| | SA | 7 | .758 | .567-1.173 | |

Whitney-Mann U test followed by Fisher's Exact test was applied to assess group differences. Two-tailed *P*-values are depicted and comparisons that were statistically significant are highlighted in gray. MDD, major depressive disorder; IQR, interquartile range; SA, suicide attempt.

**Supplemental Table 3: Results of transcription factor (TF) binding prediction using Regnetwork TF-miRNA database.**

| TF symbol | miRNA | miRNA ID | confidence |
|---|---|---|---|
| BMP4 | hsa-miR-200a | MIMAT0000682 | high |
| GATA3 | hsa-miR-200a | MIMAT0000682 | high |
| GEMIN2 | hsa-miR-200a | MIMAT0000682 | high |
| HDAC4 | hsa-miR-200a | MIMAT0000682 | high |
| SIX1 | hsa-miR-200a | MIMAT0000682 | high |
| SMAD3 | hsa-miR-200a | MIMAT0000682 | high |
| SNAI2 | hsa-miR-200a | MIMAT0000682 | high |
| TGFB1 | hsa-miR-200a | MIMAT0000682 | high |
| TP53 | hsa-miR-200a | MIMAT0000682 | high |
| TWIST1 | hsa-miR-200a | MIMAT0000682 | high |
| ZEB1 | hsa-miR-200a | MIMAT0000682 | high |
| ZEB2 | hsa-miR-200a | MIMAT0000682 | high |
| EGR1 | hsa-miR-30a | MIMAT0000087 | high |
| ESR2 | hsa-miR-30a | MIMAT0000087 | high |
| EGR1 | hsa-miR-30e | MIMAT0000692 | high |

**Supplementary Table 4: Common predicted targets of the miRNA-30a, miRNA-30e, and miRNA-200a**

| Target gene | Ensembl ID | Gene description |
|---|---|---|
| MBNL1 | ENSG00000152601 | muscleblind like splicing regulator 1 |
| RWDD4 | ENSG00000182552 | RWD domain containing 4 |
| DNAJC13 | ENSG0000013824 | DnaJ heat shock protein family (Hsp40) member C13 |
| TMEM170B | ENSG0000020526 | transmembrane protein 170B |
| HOXA11 | ENSG00000005073 | homeobox A11 |
| TTLL7 | ENSG00000137941 | tubulin tyrosine ligase like 7 |
| PTP4A1 | ENSG00000112245 | protein tyrosine phosphatase 4A1 |
| GNA13 | ENSG00000120063 | G protein subunit alpha 13 |
| ARL4A | ENSG00000122644 | ADP ribosylation factor like GTPase 4A |
| CALCR | ENSG00000004948 | calcitonin receptor |
| CCNE2 | ENSG00000175305 | cyclin E2 |
| B3GNT5 | ENSG00000176597 | UDP-GlcNAc:betaGal beta-1,3-N-acetylglucosaminyltransferase 5 |
| REEP3 | ENSG00000165476 | receptor accessory protein 3 |
| CFL2 | ENSG00000165410 | cofilin 2 |
| RARB | ENSG00000077092 | retinoic acid receptor beta |
| RAB38 | ENSG00000123892 | RAB38, member RAS oncogene family |
| ERG | ENSG00000157554 | ETS transcription factor ERG |
| STX2 | ENSG00000111450 | syntaxin 2 |
| YPEL5 | ENSG00000119801 | yippee like 5 |
| TRIM23 | ENSG00000113595 | tripartite motif containing 23 |

ADP, adenosine diphosphate; betaGal, beta-galactosidase; ETS, erythroblast transformation specific; GlcNAc, *N*-acetylglucosamine; GTP, guanosine triphosphate; Hsp40, heat shock protein 40; RAB, ras-associated binding; RAS, rat sarcoma; UDP, uridine diphosphate.
